# Gene Expression Study of Monocytes/Macrophages during Early Foreign Body Reaction and Identification of Potential Precursors of Myofibroblasts

**DOI:** 10.1371/journal.pone.0012949

**Published:** 2010-09-23

**Authors:** Lindsay Mesure, Geofrey De Visscher, Ilse Vranken, An Lebacq, Willem Flameng

**Affiliations:** Laboratory of Experimental Cardiac Surgery, Department of Cardiovascular Diseases, KULeuven, Leuven, Belgium; Universidade do Porto, Portugal

## Abstract

Foreign body reaction (FBR), initiated by adherence of macrophages to biomaterials, is associated with several complications. Searching for mechanisms potentially useful to overcome these complications, we have established the signaling role of monocytes/macrophages in the development of FBR and the presence of CD34^+^ cells that potentially differentiate into myofibroblasts. Therefore, CD68^+^ cells were *in vitro* activated with fibrinogen and also purified from the FBR after 3 days of implantation in rats. Gene expression profiles showed a switch from monocytes and macrophages attracted by fibrinogen to activated macrophages and eventually wound-healing macrophages. The immature FBR also contained a subpopulation of CD34^+^ cells, which could be differentiated into myofibroblasts. This study showed that macrophages are the clear driving force of FBR, dependent on milieu, and myofibroblast deposition and differentiation.

## Introduction

Implanted biomaterials, usually physically and chemically stable, non-immunogenic and non-toxic, trigger an innate immune response. This foreign body reaction (FBR) is associated with several complications for implanted medical devices [1]–[3]. FBR is initiated by the adhesion and denaturation of fibrinogen (FG) [4] exposing P1 and P2 epitopes recognized by Mac-1 integrin of phagocytes (neutrophils and macrophages) [5]. Degradable implant materials are removed by macrophages, while non-degradable implant materials will be encapsulated [6]. This FBR capsule comprises 1 internal layer of macrophages, several layers of (myo)fibroblasts and, in the case of intraperitoneal implantation, an external layer of mesothelial cells [7]. However, to do so the macrophage should alter its classical phagocytic action, hypothetically to more wound healing phenotype, thereby providing the following functions: inhibition of pro-inflammatory cytokine production, promotion of extracellular matrix deposition, attraction of other immune cells.

Recently, we showed attraction of different types of primitive cells, possessing colony forming capacity and differentiation potential towards the adipo-, osteo- and myofibroblast lineages, during the immature stages of the FBR [8]. These cells might therefore be the progenitors of FBR's (myo)fibroblast population. Yet, macrophages remain present during the different stages of FBR [8] and might also transdifferentiate into myofibroblasts [9]–[11]. Furthermore, it has been found that fibrocytes, a specific macrophage-derived population, contribute to wound healing and other myofibroblast tissue formations [12]–[16]. Nevertheless, too little is known about the signaling mechanisms of macrophages in the FBR which could be involved in attracting the primitive cells.

This study profiles gene expression of in vitro fibrinogen (FG) activated monocytes/macrophages as well as that of freshly isolated CD68^+^ cells from 3-days implants, against a background of unactivated monocytes/macrophages. We aimed at distinguishing between 3 major responses. The first or adhesion response is the altered gene expression induced by binding of denatured fibrinogen solely. The FBR response appears when the monocyte/macrophage is in its natural FBR environment in vivo. Finally, the adhesion in FBR response defined as the overlap between both the adhesion and FBR response.

Another aim was to functionally examine the isolated CD68^+^ and CD34^+^ populations and assess their myofibroblast differentiation potential. This was all done at the very early stage of FBR, well before the in vivo appearance of myofibroblast markers in this tissue, but at a stage where recruitment and signaling seem to be crucial [8].

## Materials and Methods

### Animals and ethics statement

Male Wistar rats (380–400g) were selected, for gene expression (n = 27) and in vitro cell studies (n = 24), and cared for in accordance with the “Guide for the Care and Use of Laboratory Animals” (NIH publication 85-23, revised 1985). The study was approved by the ethics committee for animal experiments of the Catholic University of Leuven, Belgium, on 18 March 2008 (P043/2008).

### Surgery

Anaesthesia was induced with 4% isoflurane in 100% oxygen delivered at 1 l/min for 5 min and maintained with 2% isoflurane in 100% oxygen delivered at 0.5 l/min during the surgical procedure taking approximately 20 min. After shaving and disinfecting the abdomen, a pararectal incision of approximately 1.5 cm was made through the skin, abdominal muscles and peritoneum. Acellular photo-oxidized bovine pericardium patches (1.3 cm^2^) (Cardiofix™, Sulzer Carbomedics, Austin, Texas, USA), suspended in stainless steel cages, were inserted into the peritoneal cavity and fixed to the abdominal wall with transabdominal sutures.

### Cell isolation

After 3 days in the peritoneal cavity, deposited neotissue was harvested from the retrieved patches and incubated in a 0.2% collagenase A (Roche Diagnostics, Indianapolis, USA)/0.3% plasmin (Sigma-Aldrich, Bornem, Belgium) solution for 30 min at 37°C. The suspension was then poured over a 100 µm and 40 µm cell strainer (BD Biosciences, Erembodegem, Belgium) and the red blood cells (RBC) were lysed with 10 ml 100 mM NH_4_Cl (Sigma-Aldrich). Subsequent steps were performed at 4°C. The cells were washed with PBS and labeled with a biotinylated CD68 (clone ED1; Serotec, Oxford, UK) or CD34 antibody (Clone QBEnd 10; Dako, Heverlee, Belgium) and subsequently with anti-biotin or anti-mouse paramagnetic microbeads (Miltenyi Biotec GmbH, Bergisch Gladbach, Germany), respectively. The cell suspension was applied onto a MACS separation column (Miltenyi Biotec) and the CD68^+^ or CD34^+^ cells were again poured over a new MACS separation column to enhance specific selection. Subsequently, from the CD34^−^ cell fraction, the CD68^+^ cells were collected. Immediately after isolation of CD68^+^ cells, the cells were exposed to polystyrene SPHERO Fluorescent Yellow Particles of 0.87 µm (Spherotech Inc., IL, USA) at a concentration of 20 beads/cell for 1 hour at 37°C in a volume of 100 µl PBS. Phagocytosis of the beads was confirmed by an Axiocam MRc5 camera (Zeiss).

### Cell culture

CD68^+^, as well as CD34^+^, CD34^−^CD68^+^ and CD34^−^CD68^−^ cells isolated from 3-days implants were cultured in Mesencult medium containing 20% mesenchymal stem cell stimulatory supplements (StemCell Technologies, Vancouver, Canada) and 1% antibiotics (Invitrogen, Merelbeke, Belgium). Cultures (10^5^ cells/cm^2^) were performed in 8-chamber polystyrene vessels (BD Biosciences) for 1–3 weeks.

### *In vitro* activation

Heparinized blood, collected by cardial punction from anaesthetized rats, was suspended in 1 volume PBS (pH 7.4, Invitrogen), applied onto the same volume of Histopaque 1077 (Sigma-Aldrich) and centrifuged (750×*g*) for 30 min. Mononuclear cells were collected and washed with PBS. RBCs were lysed and CD68^+^ cells purified as aforementioned. Selected cells were resuspended in RPMI 1640 culture medium (Sigma-Aldrich), with 2 mM L-glutamine (Invitrogen), 1% antibiotics or antimycotic solution (Invitrogen) and 10% foetal bovine serum (Invitrogen), and seeded at a concentration of 200.000 cells/well onto PTFE (Gore-Tex® Vascular Graft, Gore, Flagstaff, Arizona) coated with FG (10 µg/ml) (Labconsult NV, Brussels, Belgium) in a 24 well plate and cultured for 3 days. The optimal coating concentration (data not shown) was validated by ELISA with an anti-fibrinogen antibody (Gentaur, Brussels, Belgium). Monocytes/macrophages purified from blood by the aforementioned CD68^+^ selection, without in vitro incubation, served as controls.

### Gene expression

Total RNA was isolated from FG-activated, FBR and blood CD68^+^ cells using Trizol (Invitrogen) and RNeasy Mini RNA isolation columns (Qiagen, Hilden, Germany).

For microarray processing, in vitro activated, FBR and blood CD68^+^ cells were analyzed in triplicate on Rat Genome 230 2.0 Array GeneChips (Affymetrix, High Wycombe, UK) by a specialized microarray facility (DNAVision, Gosselies, Belgium) according to the manufacturer's double-round T7-based amplification protocol (Affymetrix). The data discussed in this publication have been deposited in NCBI's Gene Expression Omnibus [17] and are accessible through GEO Series accession number GSE21682 (GSM540894-GSM540902) (http://www.ncbi.nlm.nih.gov/geo/query/acc.cgi?acc=GSE21682).

The Microarray Suite Statistical Algorithm (MAS5.0, Affymetrix) was applied for analysis. For comparison analysis, the contrasts were computed with the Robust Multichip Analysis (RMA) algorithm using the R statistical language [18]. Gene expression data were visualized in the context of specific biological and functional pathways using Gene Map Annotator and Pathway Profiler (GenMAPP, http://genmapp.org). The contribution of overexpressed genes (p<0.001) in the different pairwise comparisons was investigated with GenMAPP 2.1 (www.genmapp.org) in 31 out of 149 available pathway MAPPs (www.wikipathways.org) (May 2010) [19], related to signaling and immune response (Table 1). For gene expression validation, 2 PCR primer pairs (Table 2) for each of the six comparisons were designed using Primer3 shareware (http://frodo.wi.mit.edu/cgi-bin/primer3/primer3_www.cgi). RT-PCR of a standardized quantity (30 ng) of total RNA (n = 6/group) was performed as already published by our group [20]. Glyceraldehyde 3-phosphate dehydrogenase (Gapdh) gene expression was used as positive control and for normalization. 5 µl of the Gapdh PCR product and 5 µl of Mmp14 PCR product (as for the other PCR products) were mixed. One µl of each mix was analyzed using the DNA 1000 Kit with the 2100 Bioanalyzer (Agilent Technologies). Simultaneous loading allowed assessment of the genes, as already described [20].

**Table 1:** Examined GenMAPP pathways.

GenMAPP pathway	Genes overexpressed by FBR monocytes/macrophages
Alpha6-Beta4-Integrin Signaling Pathway	Cdkn1a
Apoptosis Mechanisms	Jun, Nfkbib, RGD:727889, Tnf
Apoptosis Modulation by HSP70	Hspa1a
B Cell Receptor Signaling Pathway	Atf2, Gab1, Jun, Rela
Complement Activation, Classical Pathway	/
Cytokines and Inflammatory Response (Biocarta)	Csf2, Csf3, IL-1α, IL-1β, IL-6, IL-10, Tnf
G Protein Signaling Pathways	Akap7, Slc9a1
IL-1 Signaling Pathway	IL-1α, IL-1β, IL-1r2, Irak2, Nfkbib, Rela
IL-2 Signaling Pathway	Icam1, Rela, Socs3
IL-3 Signaling Pathway	Atf2, Gab1, Socs3
IL-4 Signaling Pathway	Atf2, Dok2, IL-4rα, Rela, Socs3
IL-5 Signaling Pathway	Atf2, Icam1, Jun
IL-6 Signaling Pathway	Gab1, IL-6, Jun, Socs3
IL-7 Signaling Pathway	/
IL-9 Signaling Pathway	Socs3
Inflammatory Response Pathway	IL-4rα
Integrin-mediated Cell Adhesion	Itga5, Itgav, Mapk6
Kit Receptor Signaling Pathway	/
Matrix Metalloproteinases	Mmp13, Tnf
Monoamine GPCRs	/
Nuclear Receptors	/
Oxidative Stress	Mt1x
Peptide GPCRs	/
Signal Transduction of S1P Receptor	Mapk6
Signaling of Hepatocyte Growth Factor Receptor	Jun
Small Ligand GPCRs	Ptger2
T Cell Receptor Signaling Pathway	Jun
TGF Beta Receptor Signaling Pathway	Atf2, Cav1, Cdc27, Cdkn1a, Fosb, Jun, Tgif1
TGF Beta Signaling Pathway	Eng, Jun, Tgif1, Tnf
TNF-alpha-NF-kB Signaling Pathway	Cav1, Gab1, Nfkb2, Nfkbib, Nfkbiz, Tnf, Tnip1, Rela
Toll-like receptor signaling pathway	Cd14, Ccl4, Cxcl10, IL-1β, IL-6, Jun, Rela, Tnf

**Table 2:** Selected primers for PCR analysis.

Primer	Abbreviation	Comparison	Sequence	Product size (bp)
Glyceraldehyde 3-phosphate dehydrogenase	Gapdh	Housekeeping gene	L: AAACCCATCACCATCTTCCAR: GTGGTTCACACCCATCACAA	198
Matrix metallopeptidase 14	Mmp14	Cu - IVo	L: TACCCACACACAACGCTCACR: TCCCAAACTTATCTGGAACACC	149
Tetraspanin 5	Tspan5	Cu - IVo	L: GGGTGTTGGCATTTGTTTTCR: GGCTTGCATTGGAGTCTGTG	204
Lysosomal-associated membrane protein 3	Lamp3	Co - IVuCo - FBRu	L:AGCAGAGCATCCAGCTATCAR:ACCCACAACACAGAGGACAA	170
Neuropeptide Y	NpY	Co - IVu	L: ACTACTCCGCTCTGCGACACR: CAACGACAACAAGGGAAATG	291
Ceruloplasmin	Cp	Cu - FBRo	L: CACAGATGGCACCTTTACGAR: CAGTTTGTCATCGGCTCTTTG	223
Cathepsin L	Ctsl	Cu - FBRo	L: CAGCCTTAGCCACTCCAAAAR:CTTCTCCCACACTGCTCTCC	122
Interleukin 16	IL16	Co - FBRu	L: CAGCGAGCCTCAGAAGAAACR: TCTTCCTGTAGCAGCAGTGG	117
Chemokine (C-C motif) ligand 24	Ccl24	IVu - FBRo	L: GTGACCATCCCCTCATCTTGR: TGCTATTGCCTCGGAGTTTC	271
Vascular cell adhesion molecule 1	Vcam1	IVu - FBRo	L: GAACCCAAACAAAGGCAGAGR: TTAGCTGTCTGCTCCACAGG	169
Interleukin 18 binding protein	IL18bp	IVo - FBRu	L: GTCTCCAGCAGTCCCAACTAAR: CATTGCCCAGCCAGTAGAG	146
Transmembrane protein 97	Tmem97	IVo - FBRu	L: CTGGGCCTCTACTTCGTCTCR: TGTGAACCGCATAGATGATTG	280

Table indicating the different primers used for the PCR analysis. C = control monocytes/macrophages; IV = in vitro activated monocytes/macrophages; FBR = monocytes/macrophages derived from FBR; L = left primer; R = right primer; bp = base pairs.

### Immunohistochemistry

Prior to staining, cell cultures were fixed in ice-cold acetone for 10 minutes. Following primary antibodies were used: TGF-β receptor type II (TGF-βRII) (clone E-6; Santa Cruz Biotechnology, Santa Cruz, USA), FITC-conjugated CD34 (clone QBEnd 10; Dako), CD68 (clone ED1; Serotec), alpha smooth muscle actin (ASMA) (clone 1A4; Dako), desmin (clone D33; Serotec) and smoothelin (polyclonal; Santa Cruz Biotechnology). TGF-βRII was visualized using an Envision kit (Dako) with a horse-raddish peroxidase labeled anti-mouse antibody and amino-ethyl-carbazole as substrate. All other antibodies, except for CD34, were detected with FITC-conjugated secondary antibodies.

Simultaneous staining without primary antibodies excluded false positive results. To avoid false positive results in the ASMA staining on the freshly isolated CD68^+^ cell spots and as the mouse monoclonal CD68 antibody was already attached to the cells, these were blocked with goat anti-mouse/biotin (GAM/B) for 5 h prior to the actual staining procedure.

To detect the myofibroblast differentiation, double staining was performed after 21 days of culturing for ASMA combined with vimentin, smoothelin or desmin. After ASMA staining with a biotinylated rabbit anti-mouse secondary antibody (Dako) and streptavidin/texas red (Perkin Elmer, Waltham, USA), the second primary antibody was added and stained with a FITC-conjugated secondary antibody (Dako). Double staining for ASMA and CD68 was performed accordingly. The ASMA-CD34 double staining was executed by applying ASMA using streptavidin/texas-red and subsequent overnight staining with the CD34/FITC-conjugated primary antibody. Cell nuclei were visualized with Vectashield DAPI-containing mounting medium (Vector Laboratories, Burlingame, USA). For cell phenotyping, 500 cells in total were evaluated in each sample, as described before [21].

### Statistical analysis

Because of the confined observations, non-parametric statistics were used (SPSS 15, SPSS Inc., Chicago, IL, USA). Comparison between more than two groups was performed with a Kruskal-Wallis test. If significance (p<0.05) was observed, pairwise comparisons of the groups were performed with non-paired Wilcoxon-Mann-Whitney tests. These were considered significant if p<0.05.

## Results

### General gene expression

**Figure 1:**
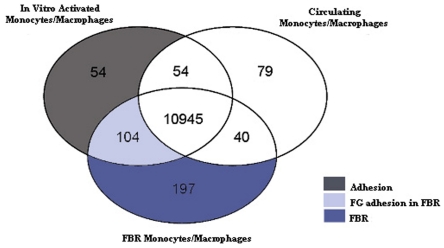
Upregulated genes. Venn-diagram showing the genetic overlap between the pairwise comparisons of in vitro activated, blood and FBR monocytes/macrophages (p<0.001).

Overall, more transcripts were present in in vitro activated and FBR CD68^+^ cells (47.1% and 45% from a total of 31099 probe sets) as compared to freshly isolated CD68^+^ cells (40.4%). Most probe sets were present (35.2%) in all samples. The distribution of gene expression comparisons (p<0.001) are summarized in a Venn-diagram (Fig. 1). The figure also clearly shows the 3 distinct groups of genes subjected to further analysis. The 54 genes overexpressed by FG-activated monocytes/macrophages compared to circulating and FBR monocytes/macrophages were considered as the adhesion response of monocytes/macrophages induced by FG activation solely. The 197 genes overexpressed by FBR CD68^+^ cells compared to the other 2 conditions represent a FBR CD68^+^ cell response, dependent on the environment. The overlap between both (104 genes) is considered FG adhesion within FBR specific genes. All upregulated genes of these 3 groups (with their associated log base 2 fold changes (logFC)) are available in supplemental tables S1, S2, and S3.

### Adhesion gene response

**Figure 2:**
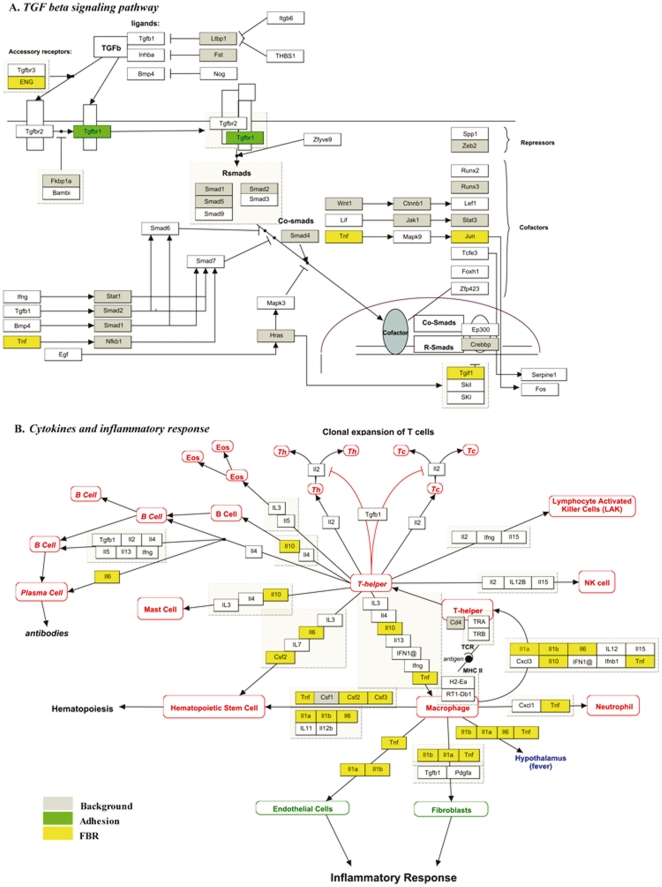
GenMAPP pathways. Upregulated genes present in GenMAPP pathways of “TGF-B signaling pathway” (A) and “cytokines and inflammatory response” (B).

Upregulated genes were found in 8 examined pathways. Epidermal growth factor receptor (EGFR) was upregulated during the primary response in the α_6_-β_4_ integrin signaling pathway of genMAPP. Whereas transforming growth factor β receptor 1 (TGF-βR1) was overexpressed in the TGF-β signaling pathway (Fig. 2A) and was together with cyclin E (Ccne1) present in the TGF-β receptor signaling pathway. FG binding of CD68^+^ cells induced upregulation of both Ccne1 and cyclin D2 (Ccnd2) in the B cell receptor signaling and only of Ccnd2 in IL-7 signaling pathway. Furthermore, somatic cytochrome c (Cycs), part of the apoptosis (apoptosis mechanisms and apoptosis modulation by HSP70) and the B-cell receptor signaling pathways, was overexpressed by in vitro activated CD68^+^ cells. Lastly, Ras protein activator like 2 (Rasal2) was apparent in the TNFα/NF-κβ signaling pathway.

### FBR gene response

All the genes exclusively overexpressed by FBR CD68^+^ cells in the 31 examined pathways are listed in table 1. This population showed upregulated expression of several cytokines and inflammatory response genes (Fig. 2B), clustered around the macrophages. These macrophages signal to T helper cells by tumor necrosis factor α (TNFα) and several interleukines (IL-1α, IL-1β, IL-6 and IL-10). These factors were also apparent in the signaling to neutrophils, fibroblasts, endothelial cells, hematopoietic stem cells and in the induction of fever. Amongst 197 genes overexpressed by these FBR monocytes/macrophages, several receptors such as intercellular adhesion molecule 1 (ICAM-1), integrin α5 (Itga5), integrin αV (ItgaV), vascular cell adhesion molecule 1 (VCAM-1), syndecan 4 (Sdc4), interleukin 4 receptor α (IL-4rα), CD14 antigen (CD14), endothelial cell adhesion molecule (Esam) were present. Matrix metalloproteinase 13 (Mmp13) was also present in this response.

### Gene response FG adhesion in FBR

FG adhesion of monocytes/macrophages during the FBR induced overexpression of disabled homolog 2 (Dab2) and activating transcription factor 3 (Atf3), which were present in the TGF-β receptor signaling pathway. While in IL-2, IL-3 and IL-6 signaling pathways, respectively phosphatidylinositol 3-kinase catalytic beta polypeptide (Pik3cb), solute carrier family 2 member 1 (Slc2a1) and serum/glucocorticoid regulated kinase (Sgk) were present. Moreover, Pik3cb is also present in the toll-like receptor and α_6_-β_4_ integrin signaling pathways. Sprouty-related EVH1 domain containing 2 (Spred2) part of the kit receptor pathway and tissue inhibitor of metallopeptidase 1 (Timp1) in the matrix metalloproteinases' pathway were also upregulated by this cell population. Lastly heme oxygenase 1 (Hmox1) was present in the oxidative stress pathway.

### PCR gene expression verification

**Figure 3:**
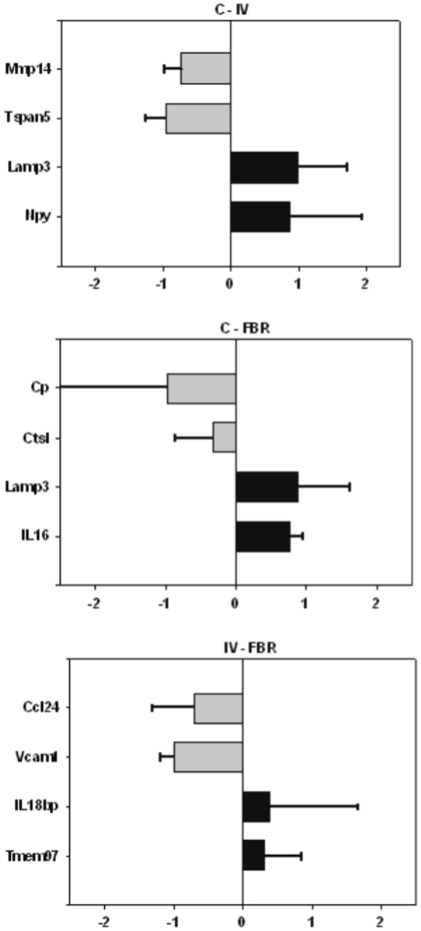
Technical verification of microarray results by RT-PCR. Overexpressed (black) and underexpressed (grey) genes are schematically represented for the 3 pairwise comparisons (A: C – IV; B: C – FBR; C: IV – FBR) by applying the “differential expression index”: (X−Y)/(X+Y) with X = mean (triplicates gene 1 in tissue type 1) and Y = mean (triplicates gene 1 in tissue type 2). Standard deviations were calculated for this differential expression index. C = control monocytes/macrophages; IV = in vitro activated monocytes/macrophages; FBR = monocytes/macrophages derived from FBR. The full gene names can be found in table 2.

The differential expression between the 3 groups of monocytes/macrophages (circulating, in vitro activated and FBR CD68^+^ cells) was technically verified by selecting 2 genes of the 10 most differentially expressed transcripts in each comparison. Reverse transcriptase-PCR results are depicted in figure 3. All the PCR products were of expected sizes and significantly confirmed the differential expression in each comparison.

### FBR cell differentiation

**Figure 4:**
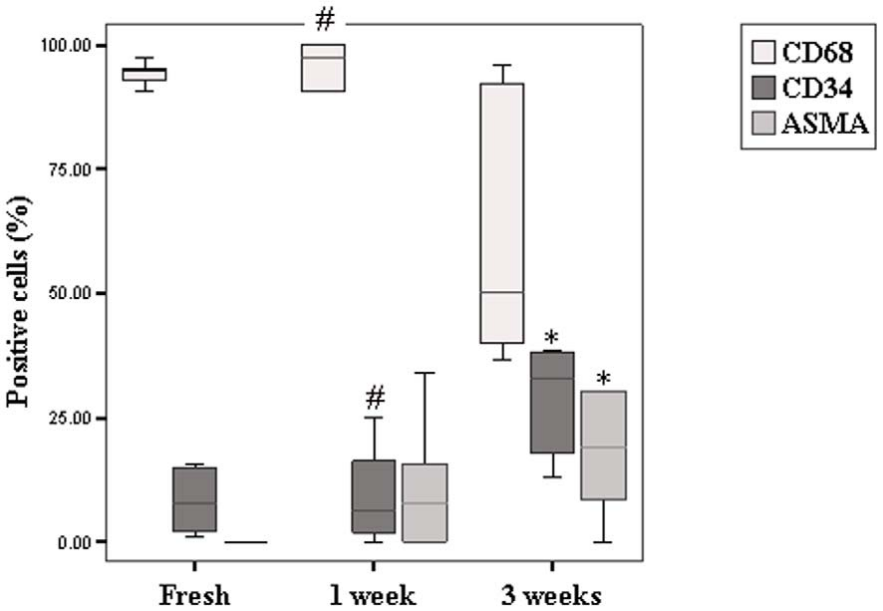
Results from immunohistochemical staining. Graph showing the percentages of CD68^+^, CD34^+^ and ASMA^+^ cells within the freshly isolated CD68^+^ cell population from the FBR as well as at 1 week and 3 weeks culturing of this CD68^+^ cell population. Bars represent mean; errors represent standard deviation. * significant difference from freshly isolated CD68^+^ cell population (FBR). # significant difference from FBR CD68^+^ cells cultured during 3 weeks.

CD68, CD34 and ASMA expression of freshly isolated FBR CD68^+^ cells compared to that of 1 week and 3 weeks cultures is summarized in figure 4. 93.6±2.7% of the cells isolated showed to be positive for the CD68 antigen, whereas 8.2±6.6% of these freshly isolated cells were CD34^+^ and no ASMA^+^ cells were present. Double staining of CD34 and CD68 was also observed (data not shown). After 1 week of culture, the CD68 and CD34 surface marker expression was comparable to that of the freshly isolated CD68^+^ cell population from the FBR. ASMA^+^ cells (10.9±13.3%) could be detected, but with high variability. Only after 3 weeks of culture, the cell population showed a significant increase in CD34 expression to the level of 28.9±11.2% (p = 0.009). ASMA expression was now significantly increased to the level of 24.7±25.2% (p = 0.015), but its high variability was maintained.

**Figure 5:**
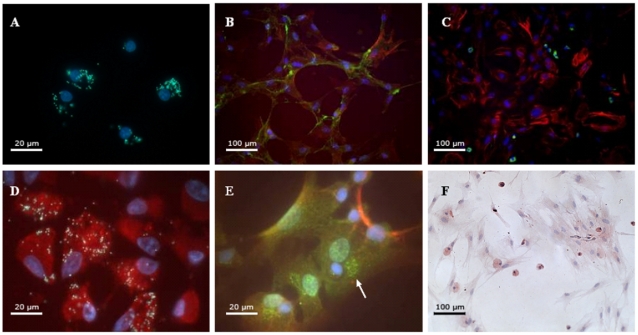
Isolation and differentiation of CD68^+^ cells. (A) Cellular phagocytosis of yellow/green fluorescent particles by the purified CD68^+^ cell population from FBR. (B) ASMA (red) and smoothelin (green) expression in FBR CD68^+^ cells cultured for 21 days in complete Mesencult medium. (C) ASMA (red) and CD68 (green) expression of isolated cells cultured for 21 days in complete Mesencult medium. Small CD68^+^ cells and larger ASMA^+^ cells can be observed. (D) Functional macrophages that have engulfed yellow fluorescent particles show an expression of ASMA (red) as well as a morphological myofibroblast aspect. (E) Double staining for ASMA (red) and CD34 (green). The presence of intracytoplasmic CD34 expression can be clearly seen (white arrow). (F) Picture showing TGF-β receptor type II staining of a 42 days old CD68+ cell culture. TGF-β receptor type II staining appears in red-brown and nuclei appear in blue. The objective lenses used are Plan-APOCHROMAT 100×/1.4 (A, D, E) and Plan-APOCHROMAT 20×/1.4 (B, C, F).

The phagocytic activity of the isolated monocyte/macrophage population (CD68^+^) was confirmed by uptake of particles (Fig. 5A and supplementary Video S1). The double staining of CD68^+^ cells cultured for 3 weeks showed the presence of vimentin, ASMA and a minimal expression of smoothelin and desmin. In figure 5B, the expression of ASMA (red) and smoothelin (green) can be observed. To verify possible transdifferentiation of ASMA^+^ cells from the CD68^+^ cell population, 21-days cultures were also double stained for ASMA and CD68. No CD68^+^ASMA^+^ cells could be observed (Fig. 5C). Yet CD68^+^ cells, that had engulfed fluorescent particles, showed ASMA expression and myofibroblast morphology with a polygonal cell shape and cell-to-cell connections after 3 weeks of culture (Fig. 5D).

In cells cultured for 21 days, no CD34 staining at membrane level could be observed. However, specifically in the ASMA^+^ cells, an intracytoplasmic CD34 staining was found (Fig. 5E). Also the CD34^+^ cell fraction showed 54.1±35.2% of ASMA^+^ cells whereas the CD34^−^CD68^+^ and CD34^−^CD68^−^ cell fractions showed 20.4±6.0% and 81.9±18.1% ASMA^+^ cells, respectively, which was a significant difference (p = 0.008). Additionally, the total CD34^+^ cell fraction, isolated from 3-days implants, showed fibrocyte-like cells after 5 days of cultivation, but no ASMA^+^ cells could be detected after 3 weeks of culture.

The possibility of autocrine stimulation with TGF-β, of which some mediators were present in the FG adhesion, FG adhesion in FBR and FBR gene responses, was also examined by determining the expression of the TGF-βRII in CD68^+^ cell cultures. As can be observed in figure 5F, expression of the TGF-β receptor type II was found in nearly all cultured cells, with stronger expression in the small round cells present.

## Discussion

With this study, we primarily aimed at understanding the functions of monocytes/macrophages in the early stages of FBR. This by studying specific gene expression profiles related to FG induced adhesion, FBR in general and FG induced adhesion of FBR monocytes/macrophages. Additionally, we have also studied the myofibroblast differentiation potential of different cell phenotypes involved in FBR.

Comparison of blood and in vitro activated monocytes/macrophages allowed identification of the primary pathways initiated by the recognition of the P1 and P2 epitope of FG. Within this adhesion response, genes were upregulated that are involved in cell adhesion (EGFR, TGF-βR1), apoptosis (Cycs), cell cycle and intracellular signaling (Ccne1, Ccnd2, Rasal2). According to the classification of macrophages into classically activated, wound-healing and regulatory macrophages, this response only showed characteristics of classically activated macrophages [22]. FG induced the upregulation of Rasal2 in the TNFα signaling pathway, which suggests already the onset of TNFα (pro-inflammatory) excretion by macrophages, which is confirmed by the expression profile of FBR CD68^+^ cells. It was already described that FG induces a pro-inflammatory response, which eventually results in the production of TGF-β [23]. This is confirmed by our study as TGF-βR1 and even TGF-β1 (p = 0.01) was upregulated in the in vitro activated monocytes/macrophages. The importance of TGF-β signaling was also apparent in the gene expression of FBR CD68^+^ cells (Fig. 2B) and even in the cell culture study (Fig. 5F).

The FBR response elucidated additional upregulation of pathways when a full FBR is induced, excluding the genes related to monocyte/macrophage specific FG adhesion analyzed separately, but including the attraction of other cells [8]. GenMAPP analysis showed the relevance of pathways related to cytokine release, (innate) immune response (IL-1α, IL-1β, IL-6, IL-10 and TNFα), cell adhesion (such as Icam-1, Vcam-1,…) and matrix remodeling (Mmp13). This response mainly showed properties of classically activated macrophages (IL-1β, IL-6, TNF, Ccl20, Cxcl10), but also of wound-healing (Factor XIII-A) and regulatory macrophages (IL-10) [22], [24]. The TGF-β production by classically activated macrophages in response to fibrinogen stimulation, can promote the alternative activation of macrophages initiated by IL-13 [23]. In this study, monocytes/macrophages of the FBR showed overexpression of IL-13 receptor alpha 1 (p = 0.004) and IL-4 receptor alpha. This was not the case in the in vitro activated CD68^+^ cells and clearly confirms the necessary interaction with the cells environment described before [22]. Moreover these FBR monocytes/macrophages showed overexpression of arginase 1 (Arg1) (p = 0.002), which can be initiated by the T_H_2 cytokines IL-4 or IL-13 and even by TGF-β or GM-CSF [25]. Arg 1 is an important enzyme in the fibroblast proliferation and in the synthesis of collagen by fibroblasts [23] and diminishes the inflammatory NO production, a function of classically activated macrophages [26].

Further analysis revealed the response of FBR monocytes/macrophages specific after binding with FG and again showed the importance of TGF-β signaling during the early FBR, as several TGF-β inducible genes (Dab2, Atf3, Timp1 and Sgk) were upregulated [27]–[30]. Heme oxygenase 1 induces the alternate activation of classically activated macrophages and confirmed the progressive switch from the pro-inflammatory to anti-inflammatory effects of macrophages during the early FBR [31].

During the FBR, primitive cells were attracted and shown to differentiate into myofibroblasts, the main cell population of mature FBR-tissue [8]. However, it has been shown that macrophages from FBR-tissue possess myofibroblast differentiation potential [9]–[11], yet they are potentially confounded with fibrocytes. Fibrocytes, of myeloid origin [32], express hematopoietic markers such as CD34, CD45 and monocyte/macrophage markers, and are known to express ASMA, vimentin and collagen upon TGF-β stimulation [15], [33].

A small fraction of the isolated cells (≈8%) expressed CD34 and might be fibrocytes, especially because signs of myofibroblast differentiation were absent. When cultured, these cells gradually expressed ASMA, as well as vimentin, smoothelin and desmin. Selective proliferation and differentiation of these cells within the CD68^+^ cell population was indicated by the decreased CD68 and increased CD34 expression during culturing.

Remarkable, there were 2 distinct populations of cells observed after 3 weeks, consisting of small, round, CD68^+^CD34^−^ASMA^−^ cells, together with very large, polygonal shaped cells being CD68^−^CD34^+^ASMA^+^. In the ASMA^+^ cells, an intracytoplasmic CD34 staining could be observed, while there was no CD34 staining in the ASMA^−^ cells. Internalization of the CD34 receptor has already been shown to exist by receptor-mediated endocytosis [34]. This intracytoplasmic CD34 staining in ASMA^+^ cells shows that these myofibroblast-like cells originate from the CD34^+^ subpopulation within the CD68^+^ cell population. This supports the fact that the CD34^+^CD68^+^ cell population is important for myofibroblast differentiation of the cultures. This cell population could also be mistaken for myeloid precursors of macrophages. So alternatively, these monocytes/macrophages attract macrophage precursors expressing C-fms and make them differentiate into myofibroblast, thus combining these findings with those of the Campbell et al. [35]. However, due to the different used techniques to isolate and describe the cells, it could also be possible that the cells in the different studies [9], [10], [13], [14] come from a common origin and only differ by their differentiation stage.

TGF-β has been shown to induce differentiation of fibrocytes [13], [15]. Autocrine TGF-β stimulation has been hypothesized to induce spontaneous differentiation of these phenotypes [15] and seems present in the CD68^+^ cell population isolated from intraperitoneal implants. This hypothesis is supported by the presence of the TGF-β receptor type II in cultured CD68^+^ cells. It was remarkable that the small round cells in culture showed much stronger signals than the larger cells, possibly because these larger, more differentiated cells didn't need signaling anymore.

Our data suggest the presence of CD34^+^ cells, possibly fibrocytes, within the purified monocyte/macrophage population. The observed relative augmentation of CD34^+^ cells in culture has already been reported for monocyte-derived fibrocyte-like cells [36]. These CD34^+^ cells actually differentiate into ASMA^+^ cells in culture as demonstrated by the internalized CD34 antigen in ASMA^+^ cells. After 14 days culturing of mononuclear cells only the fibrocyte population is still present because all other leukocytes die off [12]. Consequently, the monocyte/macrophage population from intraperitoneal implants could contain a subpopulation of fibrocyte-like cells that can survive in culture for at least 3 weeks. The CD68^+^ cells, or at least a subset thereof, are intrinsically primed to the myofibroblast differentiation pathway and spontaneously differentiate without any stimulating supplements, a property only reported for fibrocytes and probably relying on autocrine TGF-β stimulation [15]. The CD34^+^ cell fraction isolated from implants contains fibrocyte-like cells and the CD68^+^CD34^−^ cells showed no sign of myofibroblast differentiation, in contrast to the total CD68^+^ fraction. All these arguments suggest the hypothesis that the CD68^+^CD34^+^ subpopulation participate in the formation of the mature FBR-population, consisting mainly of (myo)fibroblasts. Nevertheless this remains to be confirmed by further studies.

The possibility of transdifferentiation of the total CD68^+^ cell population into ASMA^+^ cells is less likely, because no abundant presence of intermediate stages between monocytes/macrophages and myofibroblasts could be detected by the CD68/ASMA double staining. However, further studies are necessary to validate if these CD34^+^ cells actually were fibrocytes, but this was beyond the scope of this study.

In conclusion, this study shows a switch from monocytes and macrophages attracted by fibrinogen, to activated macrophages and eventually wound-healing macrophages. The 3-days immature FBR-tissue also contains a subpopulation of CD34^+^ cells capable to differentiate into myofibroblasts, the main cell population in mature FBR-tissue. Further research is necessary to clarify the effects of the detected signaling molecules on fibroblasts and define the major source of the mature FBR myofibroblasts in FBR-tissue.

## Supporting Information

Table S1 Table S1 represents all upregulated genes (p<0.001) by the adhesion of CD68+ cells to fibrinogen. C = control monocytes/macrophages; IV = in vitro activated monocytes/macrophages; FBR = monocytes/macrophages derived from FBR.
(0.07 MB DOC)

Table S2 Table S2 represents all upregulated genes (p<0.001) by the adhesion of CD68+ cells to fibrinogen during the foreign body reaction. C = control monocytes/macrophages; IV = in vitro activated monocytes/macrophages; FBR = monocytes/macrophages derived from FBR.
(0.11 MB DOC)

Table S3 Table S3 represents all upregulated genes (p<0.001) by the FBR CD68+ cells. C = control monocytes/macrophages; IV = in vitro activated monocytes/macrophages; FBR = monocytes/macrophages derived from FBR.
(0.20 MB DOC)

Video S1 Phagocytic activity of isolated CD68+ cells
(1.71 MB AVI)
