# Supplementary material for: Gene Expression Study of Monocytes/Macrophages during Early Foreign Body Reaction and Identification of Potential Precursors of Myofibroblasts
Source: PLoS One. 2010 Sep 23;5(9):e12949. doi: 10.1371/journal.pone.0012949 (PMC2944875; doi:10.1371/journal.pone.0012949)
Supplement: Table S1 — represents all upregulated genes (p<0.001) by the adhesion of CD68+ cells to fibrinogen. C = control monocytes/macrophages; IV = in vitro activated monocytes/macrophages; FBR = monocytes/macrophages derived from FBR. (0.07 MB DOC) [file pone.0012949.s001.doc]

| **Table S1**: Genes upregulated by FG adhesion (p < 0.001) | | | |
| --- | --- | --- | --- |
| ***Gene Symbol*** | ***Gene*** | ***Log FC***  ***IV-C*** | ***Log FC***  ***IV-FBR*** |
| Akr1e1 | aldo-keto reductase family 1, member E1 | 1.99 | 1.09 |
| Alcam of CD166 | activated leukocyte cell adhesion molecule | 2.8 | 3.1 |
| Apc2_predicted | adenomatosis polyposis coli 2 (predicted) | 1.53 | 1.82 |
| Ccnd2 | cyclin D2 | 3.37 | 4.64 |
| Ccne1 | cyclin E | 2.24 | 2.26 |
| Cd9 | CD9 antigen | 3.91 | 4.7 |
| Cgref1 | cell growth regulator with EF hand domain 1 | 2.4 | 2.65 |
| Cldn4 | claudin 4 | 2.66 | 2.4 |
| Clecsf6 | C-type (calcium dependent, carbohydrate recognition domain) lectin, superfamily member 6 | 5.59 | 5.03 |
| Ctsk | cathepsin K | 5.06 | 3.58 |
| Cycs | cytochrome c, somatic | 1.36 | 1.53 |
| Ddef2_predicted | development and differentiation enhancing factor 2 (predicted) | 5.32 | 4.93 |
| Dlat | dihydrolipoamide S-acetyltransferase (E2 component of pyruvate dehydrogenase complex) | 1.97 | 2.11 |
| Dst_predicted | dystonin (predicted) | 4.91 | 4.8 |
| Egfr | epidermal growth factor receptor | 4.2 | 3.78 |
| Ela1 | elastase 1, pancreatic | 3.92 | 3.4 |
| Gcsh | glycine cleavage system protein H (aminomethyl carrier) | 2.78 | 2.13 |
| Gdpd1_predicted | glycerophosphodiester phosphodiesterase domain containing 1 (predicted) | 2.95 | 2.15 |
| Got2 | glutamate oxaloacetate transaminase 2, mitochondrial | 1.61 | 1.9 |
| Gusb | glucuronidase, beta | 1.51 | 1.26 |
| Igf2r | insulin-like growth factor 2 receptor | 2.2 | 1.82 |
| Kcnn4 | potassium intermediate/small conductance calcium-activated channel, subfamily N, member 4 | 2.7 | 1.94 |
| Kif20a_predicted | kinesin family member 20A (predicted) | 2.79 | 2.41 |
| LOC291964 | similar to FH1/FH2 domain-containing protein (Formin homolog overexpressed in spleen) (FHOS) (Formin homology 2 domain-containing protein 1) | 1.93 | 1.76 |
| LOC691307 | similar to leucine rich repeat containing 39 isoform 2 | 1.41 | 1.23 |
| Melk_predicted | maternal embryonic leucine zipper kinase (predicted) | 2.32 | 2.28 |
| Minpp1 | multiple inositol polyphosphate histidine phosphatase 1 | 2.06 | 2.09 |
| Mkks | McKusick-Kaufman syndrome protein | 1.75 | 2.18 |
| Nat5_predicted | N-acetyltransferase 5 (ARD1 homolog, S. cerevisiae) (predicted) | 2.71 | 2.22 |
| Ns5atp9 | NS5A (hepatitis C virus) transactivated protein 9 | 3.8 | 3.08 |
| Osbpl2 | oxysterol binding protein-like 2 | 1.42 | 1.34 |
| Phyh2 | phytanoyl-CoA 2-hydroxylase 2 | 1.57 | 1.87 |
| Ppargc1b | peroxisome proliferative activated receptor, gamma, coactivator 1 beta | 1.63 | 2.04 |
| Ptpra | protein tyrosine phosphatase, receptor type, A | 2.21 | 1.98 |
| Rasal2_predicted | RAS protein activator like 2 (predicted) | 4.21 | 2.53 |
| RGD1307704_predicted | similar to RIKEN cDNA 2410016O06 (predicted) | 1.84 | 1.91 |
| RGD1309529_predicted | similar to DNA segment, Chr 10, ERATO Doi 214, expressed (predicted) | 1.72 | 1.95 |
| RGD1560183_predicted | similar to hypothetical protein FLJ14624 (predicted) | 3.98 | 4.56 |
| Rrm2 | ribonucleotide reductase M2 | 4.53 | 3.03 |
| Sctr | secretin receptor | 4.35 | 4.03 |
| Serping1 | serine (or cysteine) peptidase inhibitor. clade G. member 1 | 6.73 | 4.89 |
| Slc7a1 | solute carrier family 7 (cationic amino acid transporter. y+ system). member 1 | 1.98 | 2.62 |
| Solt_predicted | SoxLZ/Sox6 leucine zipper binding protein in testis (predicted) | 2.42 | 1.79 |
| Spbc25 | spindle pole body component 25 homolog (S. cerevisiae) | 2.37 | 3.44 |
| Sqle | squalene epoxidase | 1.69 | 1.65 |
| Tgfbr1 | transforming growth factor. beta receptor 1 | 2.28 | 2.8 |
| Tmem97 | transmembrane protein 97 | 1.64 | 1.57 |
| Top2a | topoisomerase (DNA) 2 alpha | 2.79 | 2.24 |
| Tyms | thymidylate synthase | 1.96 | 1.57 |
| Ube2l6 | ubiquitin-conjugating enzyme E2L 6 | 2.09 | 2.91 |
| Vnn1 | vanin 1 | 4.86 | 4.15 |
| Wfs1 | Wolfram syndrome 1 homolog (human) | 3.12 | 2.25 |
| Wrb | tryptophan rich basic protein | 2.21 | 1.81 |
| Ypel4 | yippee-like 4 | 3.7 | 3.25 |
