# Supplementary material for: Gene Expression Study of Monocytes/Macrophages during Early Foreign Body Reaction and Identification of Potential Precursors of Myofibroblasts
Source: PLoS One. 2010 Sep 23;5(9):e12949. doi: 10.1371/journal.pone.0012949 (PMC2944875; doi:10.1371/journal.pone.0012949)
Supplement: Table S2 — represents all upregulated genes (p<0.001) by the adhesion of CD68+ cells to fibrinogen during the foreign body reaction. C = control monocytes/macrophages; IV = in vitro activated monocytes/macrophages; FBR = monocytes/macrophages derived from FBR. (0.11 MB DOC) [file pone.0012949.s002.doc]

**Table S2**: Genes upregulated by FG adhesion of FBR monocytes/macrophages (p < 0.001)

| ***Gene Symbol*** | ***Gene*** | ***Log FC***  ***IV - C*** | ***Log FC***  ***FBR - C*** |
| --- | --- | --- | --- |
| Acad11_predicted | acyl-Coenzyme A dehydrogenase family. member 11 (predicted) | 3.33 | 2.41 |
| Actn1 | actinin. alpha 1 | 4.44 | 1.89 |
| Ada | adenosine deaminase | 2.08 | 2.34 |
| Adam9 | a disintegrin and metalloproteinase domain 9 (meltrin gamma) | 2.71 | 2.89 |
| Aldh7a1 | aldehyde dehydrogenase family 7. member A1 | 3.49 | 3.66 |
| Apoe | apolipoprotein E | 7.25 | 6.86 |
| App | amyloid beta (A4) precursor protein | 2.41 | 2.94 |
| Asns | asparagine synthetase | 2.56 | 1.72 |
| Atf3 | activating transcription factor 3 | 3.3 | 3.72 |
| Atp6v1b2 | ATPase. H transporting. lysosomal V1 subunit B2 | 3.06 | 2.62 |
| B4galt1_predicted | UDP-Gal:betaGlcNAc beta 1.4- galactosyltransferase. polypeptide 1 | 1.72 | 1.69 |
| B4galt6 | UDP-Gal:betaGlcNAc beta 1.4-galactosyltransferase. polypeptide 6 | 3.22 | 2.71 |
| Blvrb_predicted /// LOC681468 | biliverdin reductase B (flavin reductase (NADPH)) (predicted) /// similar to biliverdin reductase B (flavin reductase (NADPH)) | 2.4 | 3.74 |
| Camk1 | calcium/calmodulin-dependent protein kinase I | 4.14 | 3 |
| Ccrn4l /// LOC310395 | CCR4 carbon catabolite repression 4-like (S. cerevisiae) /// similar to Nocturnin (CCR4 protein homolog) | 3.51 | 5.46 |
| Cd276 | CD276 antigen | 2.54 | 2.38 |
| Cd63 | CD63 antigen | 5.27 | 4.86 |
| Chpt1 | choline phosphotransferase 1 | 4.76 | 3.49 |
| Cidea_predicted | cell death-inducing DNA fragmentation factor. alpha subunit-like effector A (predicted) | 3.86 | 3.45 |
| Clstn1 | calsyntenin 1 | 2.67 | 1.59 |
| Cltb | clathrin. light polypeptide (Lcb) | 1.46 | 1.58 |
| Cndp1 | carnosine dipeptidase 1 (metallopeptidase M20 family) | 4.37 | 5.07 |
| Ctsl | cathepsin L | 3.8 | 4.89 |
| Cyp20a1 | cytochrome P450. family 20. subfamily A. polypeptide 1 | 2.56 | 1.76 |
| Dab2 | disabled homolog 2 (Drosophila) | 4.77 | 5.16 |
| Dusp2 | dual specificity phosphatase 2 | 2.59 | 5.61 |
| Egr2 | early growth response 2 | 5.4 | 4.16 |
| Eno2 | enolase 2. gamma | 4.71 | 7.14 |
| F11r | F11 receptor | 2.87 | 1.27 |
| Fabp4 | fatty acid binding protein 4. adipocyte | 8 | 6.13 |
| Gas6 | growth arrest specific 6 | 5.55 | 4.69 |
| Hmox1 | heme oxygenase (decycling) 1 | 4.25 | 5.34 |
| Hsd3b7 | hydroxy-delta-5-steroid dehydrogenase. 3 beta- and steroid delta-isomerase 7 | 2.55 | 2.3 |
| Idh1 | isocitrate dehydrogenase 1 (NADP+). soluble | 2.67 | 1.69 |
| Il18bp | interleukin 18 binding protein | 5.12 | 1.54 |
| Jag1 | jagged 1 | 4.1 | 5.36 |
| Kdelr3_predicted | KDEL (Lys-Asp-Glu-Leu) endoplasmic reticulum protein retention receptor 3 (predicted) | 3.34 | 3.2 |
| Lhfpl2_predicted | lipoma HMGIC fusion partner-like 2 (predicted) | 2.98 | 4.63 |
| LOC362065 | CG6210-like | 2.75 | 1.86 |
| LOC367902 | similar to ALEX3 protein | 2.2 | 2.9 |
| LOC501619 | similar to 40S ribosomal protein S29 | 3.34 | 2.45 |
| LOC503000 | similar to beta-catenin-interacting protein ICAT | 2.36 | 1.48 |
| LOC679028 /// Rbpsuh_predicted | recombining binding protein suppressor of hairless (Drosophila) (predicted) /// similar to Recombining binding protein suppressor of hairless (J kappa-recombination signal binding protein) (RBP-J kappa) | 1.87 | 3.49 |
| LOC688429 | similar to Rho GTPase activating protein 10 | 3.9 | 3.25 |
| Lrp12_predicted | low density lipoprotein-related protein 12 (predicted) | 2.79 | 2.59 |
| Lxn | latexin | 2.63 | 2.25 |
| March8_predicted | membrane-associated ring finger (C3HC4) 8 (predicted) | 1.78 | 1.66 |
| Me1 | malic enzyme 1 | 3.43 | 4.28 |
| Mfsd1_predicted | major facilitator superfamily domain containing 1 (predicted) | 1.46 | 1.66 |
| MGC125167 | similar to gamma-aminobutyric acid (GABA(A)) receptor-associated protein-like 1 | 2.3 | 2.96 |
| Mrpl45_predicted | mitochondrial ribosomal protein L45 (predicted) | 1.57 | 1.12 |
| Mrvldc1 | MARVEL (membrane-associating) domain containing 1 | 2.06 | 2.91 |
| Nmnat3 | nicotinamide nucleotide adenylyltransferase 3 | 3.09 | 2.62 |
| Nudt9 | nudix (nucleoside diphosphate linked moiety X)-type motif 9 | 1.71 | 2.35 |
| Oplah | 5-oxoprolinase (ATP-hydrolysing) | 2.08 | 3.48 |
| Optn | optineurin | 3.1 | 3.11 |
| Paqr4 | progestin and adipoQ receptor family member IV | 2.34 | 1.32 |
| Pcyt1a | phosphate cytidylyltransferase 1. choline. alpha isoform | 2.87 | 2.2 |
| Pdpn | podoplanin | 2.08 | 7.09 |
| Pftk1_predicted | PFTAIRE protein kinase 1 (predicted) | 4.09 | 2.52 |
| Pik3cb | phosphatidylinositol 3-kinase. catalytic. beta polypeptide | 1.51 | 1.98 |
| Pld3 | phospholipase D family. member 3 | 5.36 | 5.64 |
| Plekhb2_predicted | pleckstrin homology domain containing. family B (evectins) member 2 (predicted) | 2.51 | 2.65 |
| Plod1 | procollagen-lysine. 2-oxoglutarate 5-dioxygenase 1 | 2.66 | 3.63 |
| Plp2 | proteolipid protein 2 | 3.24 | 2.34 |
| Pls3 | plastin 3 (T-isoform) | 3.63 | 1.23 |
| Ppap2a | phosphatidic acid phosphatase 2a | 2.22 | 2.26 |
| Prdx1 | peroxiredoxin 1 | 1.99 | 2.1 |
| Ptpmt1 | protein tyrosine phosphatase. mitochondrial 1 | 2.74 | 1.54 |
| pur-beta | transcription factor Pur-beta | 1.45 | 1.13 |
| PVR | poliovirus receptor | 3.68 | 6 |
| Rab34 | RAB34. member of RAS oncogene family | 2.94 | 2.51 |
| Rai14 | retinoic acid induced 14 | 4.21 | 2.25 |
| Rasal2_predicted | RAS protein activator like 2 (predicted) | 4.21 | 1.72 |
| RGD1303130 | kidney predominant protein NCU-G1 | 1.72 | 1.96 |
| RGD1308165_predicted | similar to hypothetical protein MGC17337 (predicted) | 2.84 | 3.59 |
| RGD1309720 | similar to Hypothetical protein MGC19163 | 2.18 | 4.24 |
| Rgl1_predicted | Ral guanine nucleotide dissociation stimulator.-like 1 (predicted) | 2.53 | 2.48 |
| Ril | reversion induced LIM gene | 2.66 | 4 |
| Scamp1 | secretory carrier membrane protein 1 | 2.5 | 2.63 |
| Scarb2 | scavenger receptor class B. member 2 | 3.91 | 3.03 |
| Sec24d_predicted | SEC24 related gene family. member D (S. cerevisiae) (predicted) | 1.89 | 2.75 |
| Sgk | serum/glucocorticoid regulated kinase | 3.92 | 4.4 |
| Slc16a1 | solute carrier family 16 (monocarboxylic acid transporters). member 1 | 2.26 | 2.61 |
| Slc22a4 | solute carrier family 22 (organic cation transporter). member 4 | 4.95 | 5.62 |
| Slc2a1 | solute carrier family 2 (facilitated glucose transporter). member 1 | 2.77 | 5.07 |
| Slc38a6 | solute carrier family 38. member 6 | 2.63 | 2.28 |
| Slco4a1 | solute carrier organic anion transporter family. member 4a1 | 4.55 | 3.08 |
| Snx24 | sorting nexing 24 | 2.04 | 1.89 |
| Spred2 | Sprouty-related. EVH1 domain containing 2 | 2.18 | 1.37 |
| ST7 | suppression of tumorigenicity 7 | 2 | 1.95 |
| Sts | steroid sulfatase | 1.84 | 3.96 |
| Surf4 | surfeit 4 | 1.99 | 2.28 |
| Tacc2 | transforming. acidic coiled-coil containing protein 2 | 3.62 | 1.88 |
| Tie1 | tyrosine kinase with immunoglobulin-like and EGF-like domains 1 | 2.03 | 1.51 |
| Timp1 | tissue inhibitor of metallopeptidase 1 | 2.88 | 4.95 |
| Tm6p1 | fasting-inducible integral membrane protein TM6P1 | 1.54 | 1.75 |
| Tmem37 | transmembrane protein 37 | 5.16 | 5.3 |
| Tnfsf9 | tumor necrosis factor (ligand) superfamily. member 9 | 2.25 | 5.32 |
| Tpi1 | triosephosphate isomerase 1 | 2 | 3.02 |
| Trp53inp2 | tumor protein p53 inducible nuclear protein 2 | 2.23 | 3.6 |
| Trpv2 | transient receptor potential cation channel. subfamily V. member 2 | 3.42 | 1.78 |
| Tspan4 | tetraspanin 4 | 3.75 | 5.04 |
| Vat1 | vesicle amine transport protein 1 homolog (T californica) | 4.56 | 4.12 |
