# Supplementary material for: Gene Expression Study of Monocytes/Macrophages during Early Foreign Body Reaction and Identification of Potential Precursors of Myofibroblasts
Source: PLoS One. 2010 Sep 23;5(9):e12949. doi: 10.1371/journal.pone.0012949 (PMC2944875; doi:10.1371/journal.pone.0012949)
Supplement: Table S3 — represents all upregulated genes (p<0.001) by the FBR CD68+ cells. C = control monocytes/macrophages; IV = in vitro activated monocytes/macrophages; FBR = monocytes/macrophages derived from FBR. (0.20 MB DOC) [file pone.0012949.s003.doc]

**Table S3**: Genes upregulated by FBR monocytes/macrophages (p < 0.001)

| ***Gene symbol*** | ***Gene*** | ***LogFC***  ***FBR - C*** | ***LogFC***  ***FBR - IV*** |
| --- | --- | --- | --- |
| Abca1 | ATP-binding cassette. sub-family A (ABC1). member 1 | 5.03 | 3.21 |
| Adm | adrenomedullin | 5.4 | 4.67 |
| Ak1 | Adenylate kinase 1 | 3.43 | 3.34 |
| Akap7 | A kinase (PRKA) anchor protein 7 | 3.23 | 2.37 |
| Aldoc | aldolase C | 3.67 | 3.22 |
| Amigo3 | amphoterin induced gene and ORF 3 | 1.11 | 1.34 |
| Aph1b | anterior pharynx defective 1b homolog (C. elegans) | 2.25 | 2.03 |
| Areg | amphiregulin | 5.53 | 4.98 |
| Arid5a | AT rich interactive domain 5A (Mrf1 like) | 3.03 | 3.9 |
| Arih1 | ariadne ubiquitin-conjugating enzyme E2 binding protein homolog 1 (Drosophila) | 1.47 | 2.18 |
| Armet_predicted | arginine-rich. mutated in early stage tumors (predicted) | 2.13 | 1.58 |
| Asgr2 | asialoglycoprotein receptor 2 | 3.72 | 4.16 |
| Atf2 | activating transcription factor 2 | 1.41 | 1.69 |
| Axud1_predicted | AXIN1 up-regulated 1 (predicted) | 3.72 | 5.86 |
| Bhlhb2 | basic helix-loop-helix domain containing. class B2 | 2.17 | 1.82 |
| Bmp2 | bone morphogenetic protein 2 | 4.47 | 3.02 |
| Bnip3 | BCL2/adenovirus E1B 19 kDa-interacting protein 3 | 5.72 | 3.44 |
| Cav1 | caveolin. caveolae protein 1 | 2.26 | 1.83 |
| Ccl20 | chemokine (C-C motif) ligand 20 | 4.02 | 3.84 |
| Ccl4 | chemokine (C-C motif) ligand 4 | 5.41 | 4.17 |
| Ccrl2_predicted | chemokine (C-C motif) receptor-like 2 (predicted) | 3.6 | 6.14 |
| Cd14 | CD14 antigen | 3.42 | 1.63 |
| Cd163_predicted | CD163 antigen (predicted) | 7.13 | 8.08 |
| Cda_predicted | cytidine deaminase (predicted) | 4.21 | 3.63 |
| Cdc27 | cell division cycle 27 homolog (S. cerevisiae) | 1.93 | 2.14 |
| Cdca4 | cell division cycle associated 4 | 2.21 | 1.96 |
| Cdkn1a | cyclin-dependent kinase inhibitor 1A | 4.99 | 2.65 |
| Clcf1 | cardiotrophin-like cytokine factor 1 | 3.39 | 5.11 |
| Cml3 | camello-like 3 | 3.15 | 2.21 |
| Crabp2 | cellular retinoic acid binding protein 2 | 3.16 | 3.37 |
| Creld1 | cysteine-rich with EGF-like domains 1 | 2.45 | 1.1 |
| Crem | CAMP responsive element modulator | 2.27 | 2.92 |
| Cryga | crystallin. gamma A | 2.36 | 2.07 |
| Csf2 | colony stimulating factor 2 (granulocyte-macrophage) | 4.94 | 4.83 |
| Csf3 | colony stimulating factor 3 (granulocyte) | 5.57 | 4.95 |
| Ctsc | cathepsin C | 2.51 | 1.15 |
| Cxcl10 | chemokine (C-X-C motif) ligand 10 | 4.3 | 5.34 |
| Dgat2 | diacylglycerol O-acyltransferase homolog 2 (mouse) | 4.23 | 3.57 |
| Dok2_predicted | docking protein 2 (predicted) | 2.51 | 1.19 |
| Dot1l_predicted | DOT1-like. histone H3 methyltransferase (S. cerevisiae) (predicted) | 3.7 | 2.76 |
| Dusp16_predicted | dual specificity phosphatase 16 (predicted) | 2.45 | 2.72 |
| Edn1 | endothelin 1 | 2.17 | 1.73 |
| Egln1 | EGL nine homolog 1 (C. elegans) | 3.44 | 2.95 |
| Egr1 | early growth response 1 | 4.08 | 5.99 |
| Ell2 | elongation factor RNA polymerase II 2 | 3.22 | 2.89 |
| Eng | endoglin | 5.31 | 2.47 |
| Epha2_predicted | Eph receptor A2 (predicted) | 1.43 | 1.56 |
| Ereg | epiregulin | 8.05 | 8.39 |
| Ero1l | ERO1-like (S. cerevisiae) | 3.88 | 3.47 |
| Errfi1 | ERBB receptor feedback inhibitor 1 | 3.06 | 3.18 |
| Esam | endothelial cell adhesion molecule | 3.31 | 2.5 |
| F13a1 | coagulation factor XIII. A1 subunit | 4.97 | 8.05 |
| F3 | coagulation factor III | 7.32 | 6.07 |
| Fam38a_predicted | family with sequence similarity 38. member A (predicted) | 2.49 | 1.72 |
| Fastk | Fas-activated serine/threonine kinase | 1.26 | 1.16 |
| Fblim1 | filamin binding LIM protein 1 | 5.07 | 2.57 |
| Fbxo30 | F-box protein 30 | 2.7 | 2.58 |
| Fkbp11 | FK506 binding protein 11 | 2.07 | 1.8 |
| Flrt3_predicted | fibronectin leucine rich transmembrane protein 3 (predicted) | 5.34 | 2.68 |
| Fmo5 | flavin containing monooxygenase 5 | 3.44 | 3.01 |
| Fosb /// Fosl2 | fos-like antigen 2 /// FBJ osteosarcoma oncogene B | 2.69 | 2.97 |
| Fstl3 | follistatin-like 3 | 4.06 | 3.32 |
| Gab1_predicted | growth factor receptor bound protein 2-associated protein 1 (predicted) | 5.54 | 4.34 |
| Gadd45a | growth arrest and DNA-damage-inducible 45 alpha | 3.68 | 6.1 |
| Gadd45b | growth arrest and DNA-damage-inducible 45 beta | 3.43 | 4.62 |
| Gdf15 | growth differentiation factor 15 | 3.44 | 2.76 |
| Gm1960 | gene model 1960. (NCBI) | 7.33 | 7.21 |
| Gpr107_predicted | G protein-coupled receptor 107 (predicted) | 1.84 | 1.55 |
| Gpr85 | G protein-coupled receptor 85 | 2.11 | 1.64 |
| Gpt1 | glutamic pyruvic transaminase 1. soluble | 3.09 | 2.44 |
| Grina | glutamate receptor. ionotropic. N-methyl D-asparate-associated protein 1 (glutamate binding) | 2.48 | 1.64 |
| Gstt1 | glutathione S-transferase theta 1 | 2.07 | 1.86 |
| H6pd_predicted | hexose-6-phosphate dehydrogenase (glucose 1-dehydrogenase) (predicted) | 3.09 | 2.51 |
| Hal | histidine ammonia lyase | 2.08 | 1.88 |
| Hdlbp | high density lipoprotein binding protein | 1.88 | 1.51 |
| Hs6st1_predicted | heparan sulfate 6-O-sulfotransferase 1 (predicted) | 1.75 | 1.44 |
| Hspa1a /// Hspa1b | heat shock 70kD protein 1A /// heat shock 70kD protein 1B (mapped) | 6.34 | 6.77 |
| Htra1 | HtrA serine peptidase 1 | 2.47 | 2.39 |
| Icam1 | intercellular adhesion molecule 1 | 4.01 | 3.27 |
| Ifrd1 | interferon-related developmental regulator 1 | 2.75 | 2.23 |
| Il10 | interleukin 10 | 5.03 | 4.94 |
| Il1a | interleukin 1 alpha | 9.31 | 7.46 |
| Il1b | interleukin 1 beta | 4.1 | 6.29 |
| Il1r2 | interleukin 1 receptor. type II | 6.16 | 4.41 |
| Il4ra | interleukin 4 receptor. alpha | 1.85 | 2.71 |
| Il6 | interleukin 6 | 6.37 | 8.75 |
| Insig1 | insulin induced gene 1 | 1.57 | 2.19 |
| Irak2 | interleukin-1 receptor-associated kinase 2 | 3.35 | 4.24 |
| Itga5 | integrin alpha 5 | 2.74 | 2.77 |
| Itgav_predicted | integrin alpha V (predicted) | 3.64 | 1.97 |
| Jun | Jun oncogene | 2.04 | 3.91 |
| Kbtbd10 | kelch repeat and BTB (POZ) domain containing 10 | 6.53 | 5.43 |
| Kdelr1 | KDEL (Lys-Asp-Glu-Leu) endoplasmic reticulum protein retention receptor 1 | 1.56 | 1.08 |
| Klf6 | Kruppel-like factor 6 | 2.49 | 3.06 |
| Kua_predicted | Kua homolog (predicted) | 2.42 | 1.79 |
| Lcn2 | lipocalin 2 | 3.21 | 3.13 |
| Lcn7 | lipocalin 7 | 2.81 | 2.19 |
| LOC314323 | transporter | 2.96 | 1.36 |
| LOC360713 /// RGD1308084 | similar to hypothetical protein FLJ11342 /// similar to pleckstrin homology-like domain. family B. member 2 | 1.5 | 1.95 |
| LOC503165 | similar to ADP-ribosylation factor GTPase-activating protein 3 (ARF GAP 3) | 2.11 | 1.24 |
| LOC679028 /// Rbpsuh_predicted | recombining binding protein suppressor of hairless (Drosophila) (predicted) /// similar to Recombining binding protein suppressor of hairless (J kappa-recombination signal binding protein) (RBP-J kappa) | 3.49 | 1.56 |
| LOC680609 /// LOC682552 | similar to Interleukin-27 beta chain precursor (IL-27B) (Epstein-Barr virus-induced gene 3 protein homolog) | 4.45 | 2.88 |
| LOC681178 /// LOC687730 | similar to polycomb group ring finger 5 | 1.48 | 1.78 |
| LOC684318 /// RGD1561481_predicted /// Usp12_predicted | ubiquitin specific protease 12 (predicted) /// similar to ubiquitin specific protease 12 (predicted) /// similar to ubiquitin specific protease 12 | 1.97 | 1.51 |
| LOC685144 | similar to SEC24 related gene family. member C | 1.46 | 1.49 |
| LOC687978 /// LOC690987 | similar to glycogen synthase 1. muscle | 3.66 | 2.66 |
| LOC687992 /// LOC691143 | similar to Serum amyloid A-3 protein precursor | 5.98 | 5.18 |
| Ltb4r | leukotriene B4 receptor | 2.92 | 3.45 |
| Mafb | v-maf musculoaponeurotic fibrosarcoma oncogene family. protein B (avian) | 3.98 | 1.67 |
| Mafg | v-maf musculoaponeurotic fibrosarcoma oncogene family. protein G (avian) | 2.01 | 1.54 |
| Map3k6_predicted | mitogen-activated protein kinase kinase kinase 6 (predicted) | 1.88 | 1.73 |
| Mapk6 | mitogen-activated protein kinase 6 | 2.56 | 2.33 |
| Mdm2_predicted | transformed mouse 3T3 cell double minute 2 homolog (mouse) (predicted) | 1.68 | 2.51 |
| Mmp13 | matrix metallopeptidase 13 | 3.03 | 3.05 |
| Mt1a | metallothionein 1a | 2.56 | 2.72 |
| Mtmr7_predicted | myotubularin related protein 7 (predicted) | 2.68 | 3.17 |
| Myo9b | myosin IXb | 1.2 | 1.44 |
| Ncf1 | neutrophil cytosolic factor 1 | 2.05 | 1.89 |
| Nfat5_predicted | nuclear factor of activated T-cells 5 (predicted) | 1.21 | 3.37 |
| Nfkb2 | nuclear factor of kappa light polypeptide gene enhancer in B-cells 2. p49/p100 | 2.94 | 3.82 |
| Nfkbib | nuclear factor of kappa light chain gene enhancer in B-cells inhibitor. beta | 3.06 | 3.02 |
| Nfkbiz_predicted | nuclear factor of kappa light polypeptide gene enhancer in B-cells inhibitor. zeta (predicted) | 1.74 | 5.04 |
| Nol3 | nucleolar protein 3 (apoptosis repressor with CARD domain) | 2.3 | 1.19 |
| Nos2 | nitric oxide synthase 2. inducible | 6.49 | 6.46 |
| Nrg1 | neuregulin 1 | 5.84 | 5.31 |
| P7 | P7 protein | 1.58 | 1.68 |
| Pdk1 | pyruvate dehydrogenase kinase. isoenzyme 1 | 3.43 | 2.4 |
| Pdlim7 | PDZ and LIM domain 7 | 2.32 | 2.72 |
| Pfkl | phosphofructokinase. liver. B-type | 4.07 | 2.71 |
| Phlda1 | pleckstrin homology-like domain. family A. member 1 | 3.66 | 3.09 |
| Pim3 | serine/threonine-protein kinase pim-3 | 2.75 | 2.52 |
| Plcb4 | phospholipase C. beta 4 | 2.31 | 3.22 |
| Plod2 | procollagen lysine. 2-oxoglutarate 5-dioxygenase 2 | 4.81 | 3.13 |
| Plscr1 | phospholipid scramblase 1 | 3.99 | 2.9 |
| Ppm1a | protein phosphatase 1A. magnesium dependent. alpha isoform | 2.09 | 1.82 |
| Ppp1r3b | protein phosphatase 1. regulatory (inhibitor) subunit 3B | 5.08 | 2.84 |
| Prkaa1 | protein kinase. AMP-activated. alpha 1 catalytic subunit | 1.29 | 1.62 |
| Ptger2 | prostaglandin E receptor 2. subtype EP2 | 4.01 | 1.98 |
| Ptgs2 | prostaglandin-endoperoxide synthase 2 | 8.22 | 9.16 |
| Ptpn1 | protein tyrosine phosphatase. non-receptor type 1 | 2.39 | 2.93 |
| Ralgds | ral guanine nucleotide dissociation stimulator | 3.44 | 2.65 |
| Rapgef2_predicted | Rap guanine nucleotide exchange factor (GEF) 2 (predicted) | 3.96 | 3.36 |
| Rela | v-rel reticuloendotheliosis viral oncogene homolog A (avian) | 1.9 | 2.38 |
| Rgc32 | response gene to complement 32 | 4.05 | 1.85 |
| RGD1303232 | Phytn_dehydro and Pyr_redox domain containing protein RGD1303232 | 1.12 | 1.26 |
| RGD1305778 | similar to RIKEN cDNA 4933405A16 | 2.47 | 1.48 |
| RGD1307401 | similar to RIKEN cDNA 2310005P05 | 4.15 | 3.12 |
| RGD1309871_predicted | similar to RIKEN cDNA 5730596K20 (predicted) | 1.92 | 1.86 |
| RGD1310953 | similar to RIKEN cDNA 2610510J17 | 3.35 | 2.28 |
| RGD1359509 | similar to hypothetical protein FLJ13448 | 2.4 | 2.89 |
| RGD1359713 | hypothetical RNA binding protein RGD1359713 | 4.33 | 1.64 |
| RGD1559442_predicted | similar to SET binding factor 2 (predicted) | 1.59 | 2.26 |
| RGD1559673_predicted | similar to hypothetical protein PP1665 (predicted) | 3.17 | 2.15 |
| RGD1559716_predicted | similar to protein kinase/endoribonuclease(IRE1) alpha (predicted) | 1.01 | 1.47 |
| RGD1559968_predicted | Similar to ADP-ribosylation factor guanine nucleotide factor 6 isoform a (predicted) | 3.39 | 3.38 |
| RGD1562047_predicted | similar to Cyclin-dependent kinases regulatory subunit 2 (CKS-2) (predicted) | 4.81 | 3.73 |
| RGD1564403_predicted | similar to Leucine rich repeat and sterile alpha motif containing 1 (predicted) | 1.69 | 1.59 |
| RGD1564480_predicted /// Smox_predicted | spermine oxidase (predicted) /// similar to polyamine oxidase isoform 2 (predicted) | 2.75 | 2.48 |
| RGD1564681_predicted | similar to mitochondrial ribosomal protein S24 (predicted) | 2.89 | 1.88 |
| RGD1566394_predicted | similar to cysteine-rich glycoprotein (predicted) | 3.46 | 3.91 |
| Rhbdl6_predicted | rhomboid. veinlet-like 6 (Drosophila) (predicted) | 1.63 | 2.47 |
| Rnd1 | Rho family GTPase 1 | 3.52 | 3.55 |
| Sdc4 | syndecan 4 | 6.56 | 4.06 |
| Sh3px3_predicted | SH3 and PX domain containing 3 (predicted) | 2.25 | 1.47 |
| Siah2 | seven in absentia 2 | 1.53 | 2.31 |
| Sipa1l2 | signal-induced proliferation-associated 1 like 2 | 2.71 | 2.15 |
| Slc30a7 | solute carrier family 30 (zinc transporter). member 7 | 2.45 | 1.49 |
| Slc36a2 | tramdorin 1 | 4.11 | 3.47 |
| Slc39a6 | solute carrier family 39 (metal ion transporter). member 6 | 1.58 | 1.69 |
| Slc41a1_predicted | solute carrier family 41. member 1 (predicted) | 2.16 | 2.2 |
| Slc7a8 /// Syngap1 | solute carrier family 7 (cationic amino acid transporter. y+ system). member 8 /// synaptic Ras GTPase activating protein 1 homolog (rat) | 3.32 | 1.85 |
| Slc9a1 | solute carrier family 9. member 1 | 1.82 | 1.61 |
| Slk | serine/threonine kinase 2 | 2.3 | 1.47 |
| Smox_predicted | spermine oxidase (predicted) | 2.75 | 2.6 |
| Snag1_predicted | sorting nexin associated golgi protein 1 (predicted) | 1.94 | 1.51 |
| Socs3 | suppressor of cytokine signaling 3 | 2.36 | 2.5 |
| Stc2 | stanniocalcin 2 | 2.82 | 2.79 |
| Stch | stress 70 protein chaperone. microsome-associated. 60kD human homolog | 2.1 | 1.49 |
| Stx11 | syntaxin 11 | 2.66 | 3.78 |
| Stxbp5 | syntaxin binding protein 5 (tomosyn) | 2.86 | 2.75 |
| Tfec | transcription factor EC | 5.07 | 3.32 |
| Tgif | TG interacting factor | 1.54 | 1.48 |
| Tle4 | transducin-like enhancer of split 4. E(spl) homolog (Drosophila) | 1.92 | 2.93 |
| Tmem106a | transmembrane protein 106A | 2.91 | 2.57 |
| Tmem39a | transmembrane protein 39a | 1.61 | 1.59 |
| Tmem55a | transmembrane protein 55A | 2.97 | 1.17 |
| Tnf | tumor necrosis factor (TNF superfamily. member 2) | 5.58 | 5.04 |
| Tnip1_predicted | TNFAIP3 interacting protein 1 (predicted) | 2.62 | 2.48 |
| Trib1 | tribbles homolog 1 (Drosophila) | 2.34 | 2.56 |
| Ube2q2_predicted | ubiquitin-conjugating enzyme E2Q (putative) 2 (predicted) | 1.92 | 1.22 |
| Utrn | Utrophin | 1.69 | 1.83 |
| Vcam1 | vascular cell adhesion molecule 1 | 4.79 | 4.66 |
| Vdac1 | voltage-dependent anion channel 1 | 2.36 | 1.79 |
| Vldlr | very low density lipoprotein receptor | 3.34 | 1.81 |
| Zfand2a | zinc finger. AN1-type domain 2A | 5.36 | 3.68 |
| Zfp406_predicted | zinc finger protein 406 (predicted) | 3.8 | 2.75 |
| Zfp598_predicted | zinc finger protein 598 (predicted) | 1.34 | 1.44 |
